# Evaluating Treatment Options for Opiate Use Disorder: A Meta-Analysis of Buprenorphine-Naloxone and Extended-Release Naltrexone

**DOI:** 10.7759/cureus.89769

**Published:** 2025-08-11

**Authors:** Bassam H Awaji, Sobhia N Abanmi, Manar S Alqahtani, Leen Alhodathi, Mohammed Alrehaili, Turki F Aldohan, Abdulaziz S Alkhenaizan, Zahrah M Alsughayer, Abdullah A Alyahya, Rahaf Mutab S Alanazi, Hadeel Aljohani, Hosam Hadi Hassan Awaji, Sara Y Alsuliman

**Affiliations:** 1 Otolaryngology, King Faisal Specialist Hospital and Research Centre, Riyadh, SAU; 2 College of Medicine, Majmaah University, Riyadh, SAU; 3 General Medicine, Aseer Health Cluster, Abha, SAU; 4 Medicine and Surgery, Sulaiman Al-Rajhi University, Qassim, SAU; 5 Psychology, Kingston &amp; St. Andrew Municipal Corporation, Madinah, SAU; 6 Department of Psychiatry, Dar Al Uloom University, Riyadh, SAU; 7 Medicine and Surgery, Imam Mohammad Ibn Saud Islamic University, Riyadh, SAU; 8 College of Medicine, Alrayan Colleges, Qatif, SAU; 9 College of Medicine, University of Tabuk, Tabuk, SAU; 10 General Practice, Eradah (Al Amal) Mental Health Complex, Arar, SAU; 11 Medicine and Surgery, King Abdulaziz University Faculty of Medicine, Jeddah, SAU; 12 Preventive Medicine, King Salman Armed Forces Hospital, Tabuk, SAU; 13 College of Medicine, King Saud Bin Abdulaziz University for Health Sciences, Riyadh, SAU

**Keywords:** buprenorphine, extended-release naltrexone, meta-analysis, naloxone, opioid use disorder

## Abstract

Opioid use disorder (OUD) is a chronic, relapsing condition with significant health and social consequences. Buprenorphine-naloxone (BUP-NX) and extended-release naltrexone (XR-NTX) are two primary medications used for OUD treatment. A systematic review and meta-analysis were conducted. PubMed, Embase, and Cochrane databases were searched to identify randomized controlled trials (RCTs) comparing BUP-NX and XR-NTX for OUD treatment. Studies were included based on predefined inclusion and exclusion criteria, including patient population, intervention, comparator, outcome measures, and study design. Pooled analyses were performed to assess differences in abstinence time, days of opioid use, negative urine samples, quality of life, and adverse effects. No significant difference was found between BUP-NX and XR-NTX in terms of abstinence time. However, XR-NTX demonstrated a significant advantage in reducing the number of days of opioid use. No significant differences were observed in terms of the number of negative urine samples or quality of life. Adverse effects were comparable between the two treatments. While both medications appear effective for OUD treatment, the choice between BUP-NX and XR-NTX may depend on individual patient factors. Further research is needed to clarify the long-term effects of these medications and to identify optimal treatment strategies for different patient populations.

## Introduction and background

Opiate use disorder (OUD) remains a significant public health challenge globally, with substantial morbidity, mortality, and socio-economic costs. Effective treatment strategies are crucial for improving patient outcomes and reducing the burden of this disorder. Among the pharmacological treatments available, buprenorphine-naloxone (BUP-NX) and extended-release naltrexone (XR-NTX) have emerged as prominent options [1].

The impact of OUD is profound. Individuals with OUD often experience significant impairment in their daily lives, including difficulties in maintaining employment, relationships, and overall health. The disorder is also associated with a high risk of relapse, even after periods of abstinence, making long-term management challenging. Effective treatment is essential to help individuals achieve and maintain recovery, reduce the risk of overdose, and improve quality of life [2].

BUP-NX is a combination medication used to treat OUD. Buprenorphine is a partial opioid agonist, which means it activates opioid receptors in the brain but to a lesser extent than full agonists like heroin or methadone. This helps to reduce cravings and withdrawal symptoms without producing the same level of euphoria. Naloxone is an opioid antagonist that blocks the effects of opioids and is included to prevent misuse of the medication by injection [3].

Common side effects of BUP-NX include headache, nausea, vomiting, constipation, and sweating. Some patients may experience dizziness, drowsiness, or blurred vision. Serious side effects can include respiratory depression, especially if taken in higher doses or combined with other central nervous system depressants [3].

XR-NTX is an opioid antagonist that blocks the euphoric and sedative effects of opioids. It is administered as a monthly injection, which can improve adherence compared to daily oral medications. Naltrexone works by binding opioid receptors in the brain, preventing opioids from exerting their effects. Common side effects of XR-NTX include nausea, headache, dizziness, and fatigue. Some patients may experience injection site reactions, such as pain, swelling, or redness. Serious side effects can include liver damage, which is why liver function should be monitored during treatment [4].

Despite the availability of these treatments, there is ongoing debate regarding their relative efficacy and safety. Previous studies have provided mixed results, and there is a need for a comprehensive synthesis of the existing evidence to guide clinical decision-making [5,6].

This meta-analysis aims to compare the efficacy and safety of BUP-NX versus XR-NTX in the treatment of OUD, providing a clearer understanding of their respective benefits and limitations. By systematically reviewing and analyzing data from multiple studies, this meta-analysis seeks to offer robust conclusions that can inform healthcare providers, policymakers, and patients in selecting the most appropriate treatment for OUD.

## Review

Methods

The conduction and reporting of this meta-analysis followed the principles of the Cochrane Handbook for Systematic Reviews of Interventions version 6 and the Preferred Reporting Items for Systematic Reviews and Meta-Analyses (PRISMA) guidelines [7].

Research Question

What is the comparative efficacy of BUP-NX versus XR-NTX in the treatment of opiate use disorder?

Research Aims

This study aimed to compare the efficacy of BUP-NX and XR-NTX in the treatment of OUD and to evaluate the safety profiles and side effects associated with BUP-NX and XR-NTX.

Research Objectives

The research objectives are to systematically review and synthesize existing studies comparing the efficacy of BUP-NX and XR-NTX and to analyze the incidence of side effects reported in studies involving BUP-NX and XR-NTX.

Inclusion Criteria for the Included Studies

Types of studies: This meta-analysis included randomized controlled studies that were published from inception to December 7, 2024.

Participants: Eligible studies included all studies that compared BUP-NX and XR-NTX in OUD. No restrictions were made with regard to age, sex, or race of the participants.

Interventions: Studies that were considered eligible included those that compared BUP-NX and XR-NTX in OUD.

Exclusion Criteria for the Studies

We excluded studies involving animals, retrospective analyses, conference abstracts, duplicate entries, case reports, review articles, commentaries, case series with fewer than four patients, or clinical guidelines.

Search Strategy

Electronic searches: The following electronic databases were searched for eligible studies: MEDLINE/PubMed, Cochrane Central Register of Controlled Trials (CENTRAL), Web of Science, ProQuest, and Scopus. The search was set for all articles published in English from inception till December 7, 2024.

The following search terms were used: (extended-release naltrexone) AND (buprenorphine-naloxone) AND (opioid relapse prevention). We used no filters by language or publication period. Appendix A summarizes the search terms used for each database and the count of search results.

Other resources: The first reviewer searched within the reference lists of obtained articles for other potentially relevant studies that were not retrieved by the electronic search.

Selection of Studies

The first reviewer screened the retrieved reports for eligibility through title, abstract, and full-text screening. The second reviewer checked the retrieved studies, and discrepancies were solved through discussion with a third reviewer.

Data Extraction

The first reviewer carried out data extraction from the included studies using a standardised data sheet which included (a) the study’s characteristics (author, year, country, study design); (b) patients’ characteristics (age at the time of treatment, sex, sample size); (c) intervention details (type, dose, substance use, substance route, and the duration of follow-up), and (e) the outcomes, i.e., primary outcomes (abstinence time (months) and number of negative samples) and secondary outcomes (quality of life score and adverse effects). The second reviewer checked the collected data for consistency and clarity. Any disagreements were settled by refereeing by the third reviewer.

Measured Outcomes

Primary outcomes include the abstinence time, which is calculated in months in terms of mean and SD, and the number of negative samples, i.e., mean and SD.

Secondary outcomes include the quality of life score (mean and SD) and adverse effects (event and total).

Assessment of the Risk of Bias in the Included Studies

The risk of bias (ROB) in the included studies was assessed using the National Institute for Health and Care Excellence (NICE) checklists for randomized controlled clinical trials [8].

Data Synthesis

**Figure 1:**
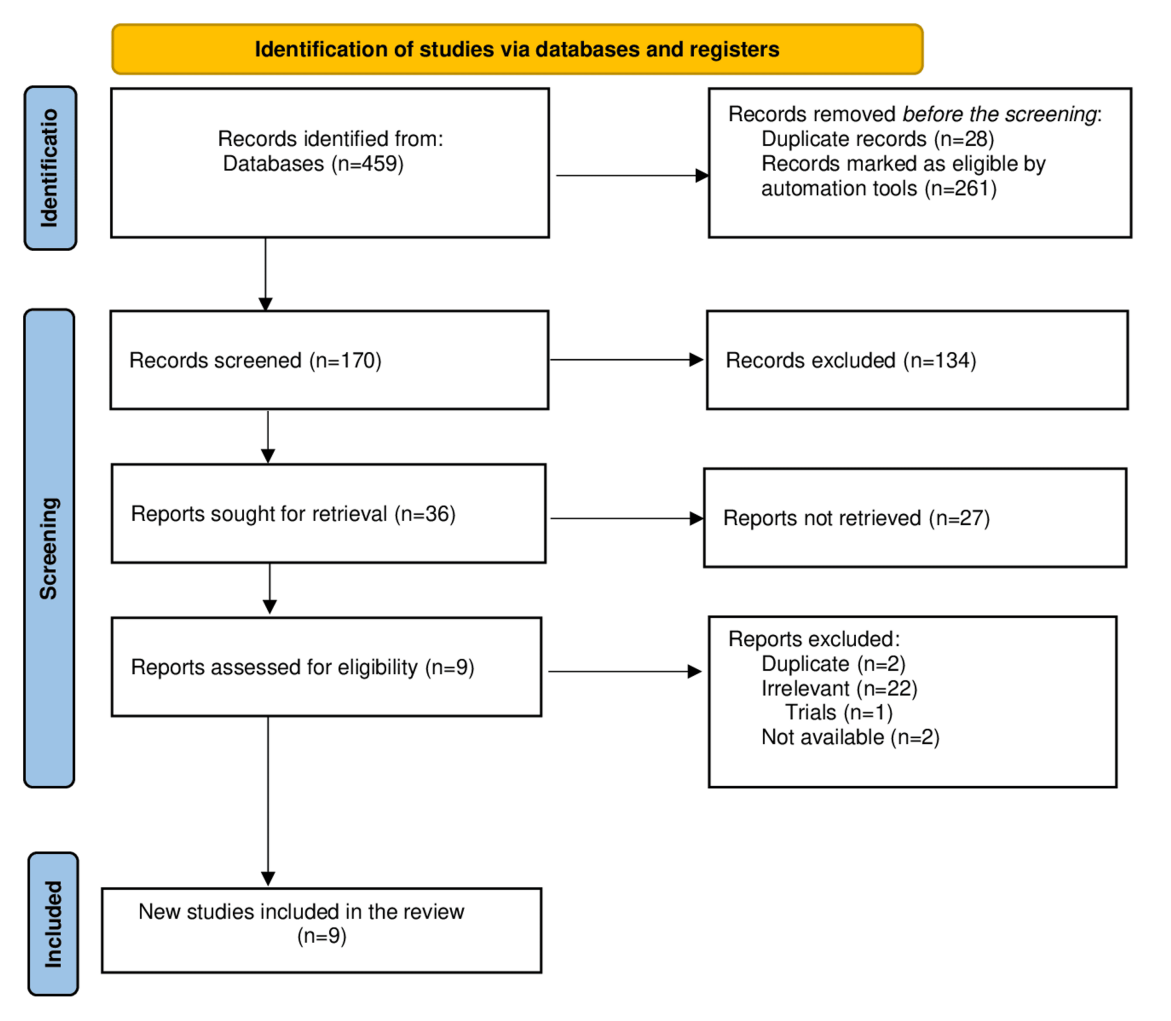
PRISMA flow chart

Initially, 459 records were retrieved from electronic database searches (Appendix A). After removing duplicates and excluded studies, 36 studies were finally eligible, of which nine studies [5,9-16] (2,399 patients) were included (Table 1), while the 27 excluded studies from the MA were either irrelevant (n = 22) or duplicate (n = 2), trials (n = 1), and not available (n = 2); these studies are mentioned in Figure 1 [17].

**Table 1:** Summary table of the included studies NM: non mentioned; BUP-NS: buprenorphine-naloxone; XR-NTX: extended-release naltrexone; RCT: randomized controlled trials

Author	Year	Country	Study design	Age		sex (M: F)		Sample size		Substance use	Substance use route	Duration of the disease	BUP‐NX dose	XR‐NTX dose	Primary outcomes	Secondary outcomes	follow up duration
				BUP‐NX	XR‐NTX	BUP‐NX	XR‐NTX	BUP‐NX	XR‐NTX								
Erdoğan et al. [5]	2022	Turkey	Retrospective cohort study	Median = 25.00 years	Median = 25.50 years	M (178), F (14)	M (194), F (14)	192	208	Heroin only: BUP-NX: 30.2%; XR-NTX: 28.8% Multiple substances: BUP-NX: 69.8%; XR-NTX: 71.2% (p = 0.765)	NM	BUP-NX group: Median = 6.00 years XR-NTX group: Median = 7.00 years	Buprenorphine‐naloxone (Suboxone®; two forms: 8/2 and 2/ 0.5 mg)	Three‐month implant XR‐NTX 1000 mg (Prodetoxone®: Subcutaneous implant NTX 1000 mg 12‐week depot form)	Abstinence time	Continuation of treatment after relapse, liver function tests	Three months
Haeny A.M. et al. [9]	2020	USA	RCT	37.9 (12.3)	40.2 (11.3)	M = 83.3%	M = 78.4%	36	37	Heroin and other illicit opioids	intravenous	NM	4-24 mg/d	380 mg	Retention in treatment, opioid-negative urine drug tests, days of use of heroin and other illicit opioids	Days of use of other illicit substances, safety assessed by adverse event reporting	Three months
Jalali A et al. [10]	2020	USA	RCT	NM	NM	NM	NM	270	204	Opioid use disorder (OUD)	Intravenous (IV) heroin use	NM	Dispensed at weeks 0, 1, 2, 3, 4, 6, 8, 10, 12, 14, 16, 20; self-administered sublingually daily; dose based on clinical indication	Intramuscular injection approximately every 28 days	Quality of life	Comparison between pharmacotherapy-responsive and characteristic-responsive classes	24-week intervention; follow-up at 28 and 36 weeks
Lee JD et al. [11]	2018	USA	RCT	33·7 (9·8	34·0 (9·5)	M (206), F (81)	M (195), F (88)	287	283	Heroin (81%)	Intravenous (63%	12.8 (9.0) years	8-24 mg/day	380 mg/month	Opioid relapse-free survival	Opioid-negative urine samples, opioid-abstinent days	Six months
Mitchell S.G. et al. [12]	2021	USA	RCT	Mean (SD) 19.4 (1.0)	19.2 (1.3)	F = 44.7%	F = 52.4%	Baseline = 93,3 months: Buprenorphine maintenance (n = 80), six months: Buprenorphine maintenance (n = 78)	Baseline = 82,3 months: Received XR-NTX (n = 66), six months: Received XR-NTX (n = 64)	Opioid use disorder (OUD)	NM	NM	12–20 mg/day	380 mg intramuscular injection (Vivitrol®), administered every four weeks	Self-reported illicit opioid use; relapse to DSM-5 defined OUD	Use of other substances (cocaine, marijuana, alcohol); adherence to treatment	Three and six months post-discharge
Murphy et al. [13]	2019	USA	RCT	33.7 (9.8)	34.0 (9.5)	M = 71.8%	M = 68.9%	287	283	NM	NM	NM	NM	NM	Incremental costs combined with incremental quality-adjusted life-years (QALYs) and incremental time abstinent from opioids	NM	Six and nine months
Rudolph et al. [14]	2021	USA	RCT	NM	NM	NM	NM	287	283	NM	NM	NM	BUP-NX: Self-administered sublingually, dispensed at specific intervals (weeks 0, 1, 2, 3, etc.). Dosing: based on clinical indication	XR-NTX: Intramuscular injections approximately every 28 days. Dosing: Based on clinical indication.	Relapse to opioid use	NM	NM
Ruglass L.M. et al. [15]	2019	USA	RCT	NM	NM	70.4% Male, 29.6% Female	70.4% Male, 29.6% Female	281	254	Opioid use disorder	Intravenous	12.5 years (mean)	8-24 mg/day	380 mg/month	Opioid use trajectories	Medical management visits, history of treatment succes	Six months
Tanum et al. [16]	2017	Norway	RCT	35.7 (8.5)	36.4 (8.8)	M (54), F (25)	M (61), F (19)	79	82	Heroin and other illicit opioids	Intravenous	NM	4 to 24 mg/d	380 mg every fourth week	Completion rate, opioid-negative urine drug tests, days of heroin and other illicit opioid use	Days of use of other illicit substances	Three months

Statistical Analysis

Meta-analysis was performed using Review Manager (RevMan) version 5.4 [18]. Pooled mean differences (MDs) were used for comparing the treatment control of opioid use, such as the abstinence days, number of days of using the opioid drugs while on treatment, number of negative urine samples among the participants, and improvement of QOL scores. Odds ratio (OR) was used to compare the rate of adverse effects between the treatment arms. The pooled studies with 95% confidence intervals (CIs) were calculated using a random-effects model due to expected heterogeneity among studies. Heterogeneity was assessed using the I² statistic, with values above 50% indicating substantial heterogeneity. Sensitivity analyses were conducted by excluding studies one by one to assess the robustness of the results.

Results

This meta-analysis compares the efficacy of BUP-NX and XR-NTX. Nine studies with 2,399 patients were finally eligible for analysis [5,9-16].

Primary Outcomes

**Figure 2:**
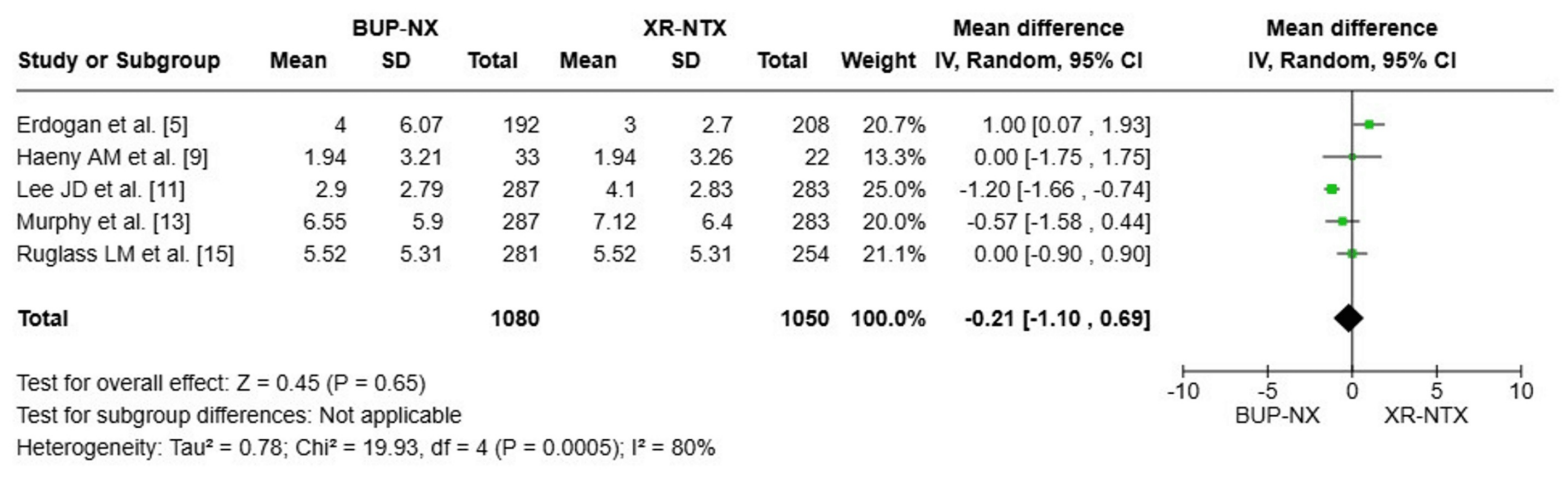
Forest plot compares the abstinence time (in months) in the two treatment groups

Control of opioid use (abstinence time (in months)): In terms of abstinence time (months) in individuals with opiate use disorder. The pooled analysis includes five studies (n = 2,130) [5,9,11,13,15]. The overall mean difference (MD) was -0.21 months (95% CI: -1.10 to 0.69, P = 0.65), indicating no statistically significant difference between the two treatments. The analysis showed substantial heterogeneity (I² = 80%), suggesting variability among the included studies. The heterogeneity might be resolved by excluding Lee et al. [13], which appears to contribute significantly to the observed variability (Figure 2).

**Figure 3:**
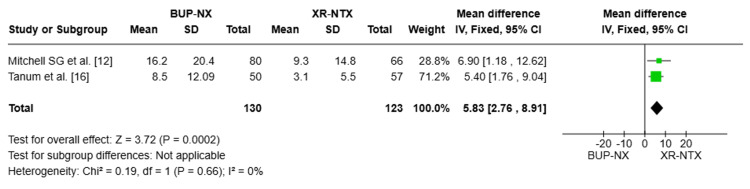
Forest plot compares number of days of opioid drugs use during the last 90 days while on treatment

Days of opioid drug use during the last 90 days while on treatment: The pooled analysis includes two studies (n = 253) [12,16]. The overall MD was 5.83 days (95% CI: (2.76 to 8.91), P = 0.0002), demonstrating a statistically significant better outcome for the XR-NTX group, with fewer days of drug use compared to BUP-NX. The analysis showed no heterogeneity (I² = 0%), indicating consistency across the included studies (Figure 3).

**Figure 4:**
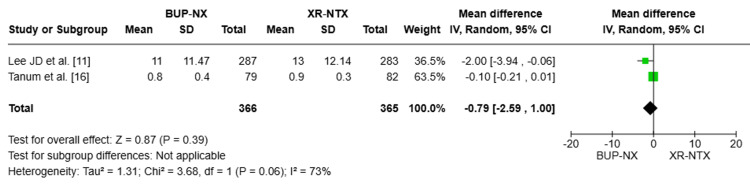
Forest plot compares the number of weeks with negative urine samples between the two groups

Number of weeks with negative urine samples: To compare the number of negative urine samples that were obtained on a weekly basis, two pooled studies were included, with a total of 731 patients [11,16]. The pooled MD was MD = -0.79, 95% CI (-2.59, 1.00), P = 0.39, indicating no statistically significant difference between the two treatments. The heterogeneity was substantial (I² = 73%), suggesting variability in study results (Figure 4).

Secondary Outcomes

**Figure 5:**
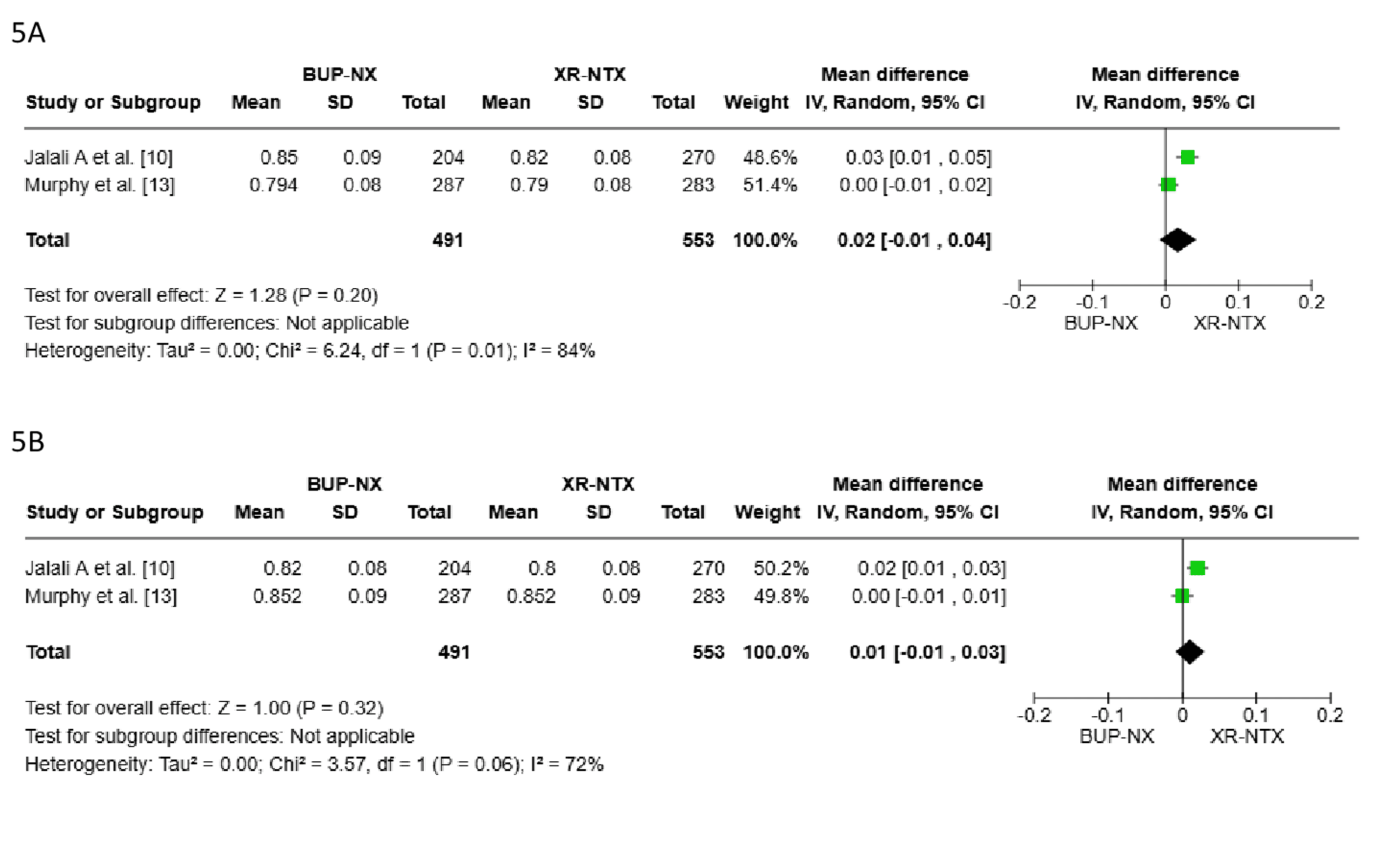
Forest plot comparison 5A: The forest plot compares the change in the quality-of-life score after six months of treatment. 5B: The forest plot compares the change in the quality-of-life score after nine months of treatment.

Quality of life improvement: In terms of improving the quality-of-life scores for individuals with OUD, two pooled studies were included, with a total of 1,044 patients [10,13]. The first analysis after six months of treatment (Figure 5A) showed an MD = 0.02, 95% CI (-0.01, 0.04), P = 0.20, indicating no statistically significant difference between the two treatments. The heterogeneity was substantial (I² = 84%), suggesting variability in the studies that could not be resolved. 

In Figure 5B, the second analysis after nine months of therapy showed an MD = 0.01, 95% CI (-0.01, 0.03), P = 0.32, indicating no statistically significant difference between the two treatments. There was substantial heterogeneity (I² = 72%) between the pooled studies, which could not be resolved [10-13].

**Figure 6:**
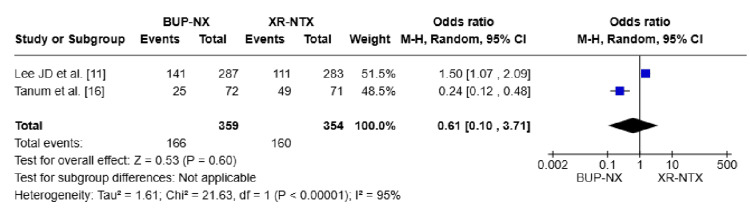
Forest plot compares the rates of adverse effects in both treatments

Adverse effects: Two pooled studies were included, involving a total of 713 participants [11-16]. The pooled OR was OR = 0.61, 95% CI (0.10, 3.71), P = 0.60, indicating no statistically significant difference in the occurrence of adverse effects between the two treatments. The heterogeneity was substantial (I² = 95%), suggesting significant variability between studies (Figure 6).

**Figure 7:**
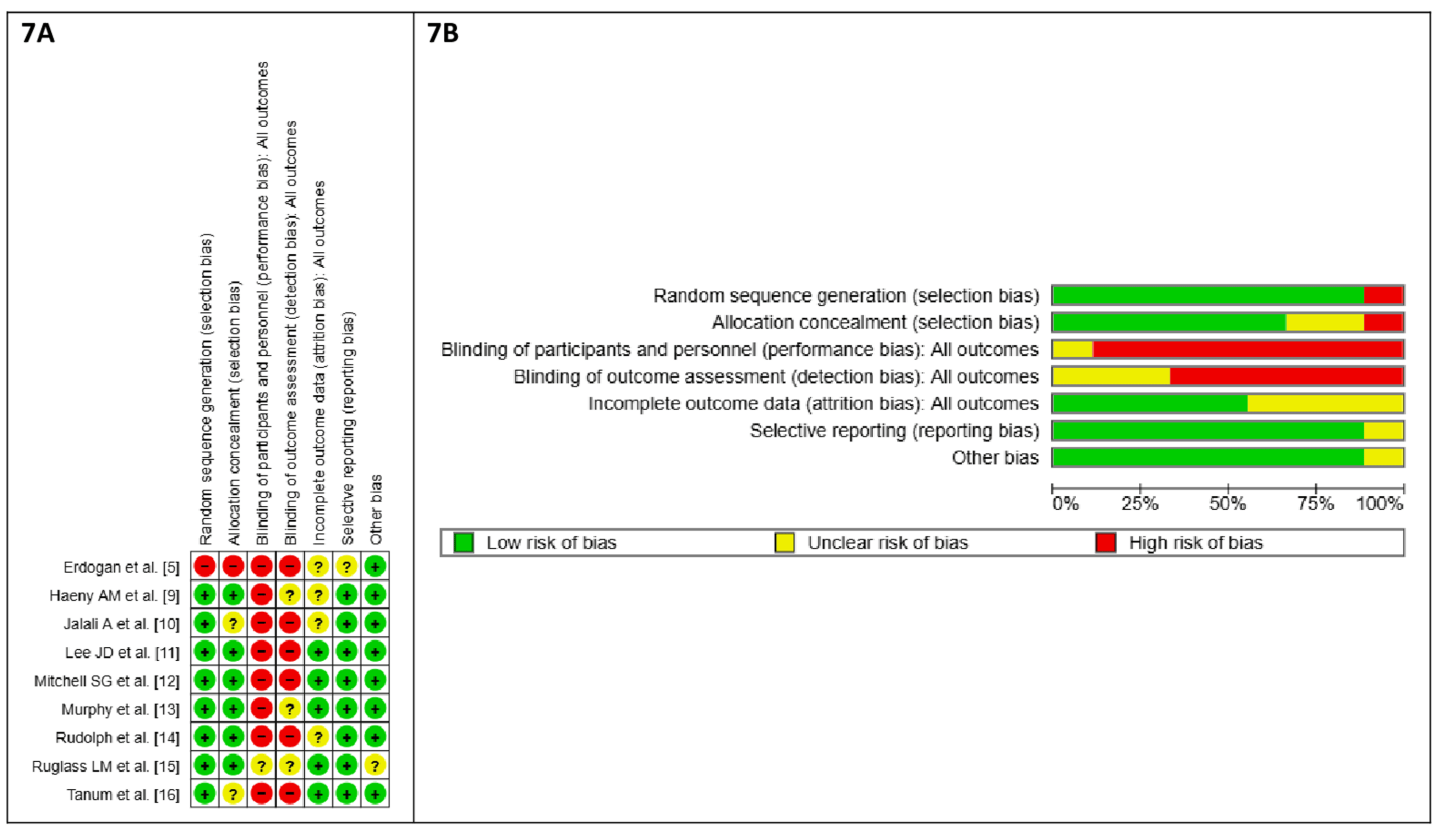
Risk-of-bias summary and graph

Risk of bias: The risk of bias for this meta-analysis was assessed across six domains. For random sequence generation (selection bias), eight out of nine studies had a low risk of bias, while one showed a high risk of bias due to a lack of randomization. Allocation concealment (selection bias) had six studies with low risk and one with high risk, while two studies did not report clear data regarding the allocation concealment. Blinding of participants and personnel (performance bias) revealed high risk in the majority of the studies, mostly due to the open-label nature of these studies and the nature of the treatments used, such as implants versus oral tablets. Regarding incomplete outcome data (attrition bias), five studies were at low risk, while four had unclear risk due to the excluded data of the drop-out patients. Selective reporting (reporting bias) showed eight studies with low risk and one with unclear risk. Finally, for other biases, all studies considered low risk except for Ruglass et al. [15], which showed unclear risk due to a lack of control for the confounders. Overall, while there were some well-conducted studies, specific domains such as allocation concealment and performance bias had notably high-risk trends (Figures 7A, 7B).

Discussion

OUD is a chronic, relapsing disease characterized by compulsive opioid use, loss of control over opioid intake, and continued use despite negative consequences. This disorder has become a significant public health crisis, leading to increased rates of overdose, addiction, and associated comorbidities [19].

Pharmacotherapy plays a crucial role in the treatment of OUD, aiming to reduce opioid craving, prevent relapse, and improve overall functioning. Two commonly used medications for OUD treatment are BUP-NX and XR-NTX. BUP-NX is a partial opioid agonist that reduces withdrawal symptoms and cravings, while XR-NTX is an opioid antagonist that blocks the euphoric effects of opioids [20].

This meta-analysis aimed to compare the effectiveness of BUP-NX and XR-NTX in treating OUD. Nine studies with a total of 2,399 participants were included in the analysis.

In terms of abstinence time, there was no statistically significant difference between the two treatments. However, the analysis showed substantial heterogeneity, indicating variability among the studies. This variability might be explained by the inclusion of one study (Lee et al.) that appeared to contribute significantly to the heterogeneity.

Regarding days of opioid drug use during the last 90 days of treatment, the results showed a statistically significant advantage for XR-NTX. Individuals receiving XR-NTX had fewer days of drug use compared to those on BUP-NX. This finding was consistent across the included studies.

When comparing the number of negative urine samples, no significant difference was found between the two treatments. However, similar to abstinence time, there was substantial heterogeneity among the studies.

For quality-of-life improvement, both after six and nine months of treatment, no statistically significant difference was observed between the two treatments. Again, substantial heterogeneity was present in both analyses.

Lastly, in terms of adverse effects, there was no significant difference between the two treatments. However, the heterogeneity was substantial, indicating significant variability among the studies.

Limitations

While some studies were well-conducted, concerns were raised about the risk of bias in specific domains, such as allocation concealment and performance bias. This was mainly due to the open-label nature of the studies and the differences in treatment administration (implants vs. oral tablets). In addition, the substantial heterogeneity in several outcomes highlights the need for caution in interpreting the results.

## Conclusions

Both BUP-NX and XR-NTX appear to be effective treatments for opioid use disorder. While XR-NTX may offer a slight advantage in reducing opioid use days, further research is needed to confirm this finding. The choice of treatment should be individualized, considering factors such as patient preference and treatment context. Future studies should focus on improving study design, particularly regarding blinding and allocation concealment, to provide more reliable and generalizable results
